# Infection of *Plasmodium falciparum* and helminths among school children in communities in Southern and Northern Ghana

**DOI:** 10.1186/s12879-021-06972-1

**Published:** 2021-12-17

**Authors:** G. Akosah-Brempong, S. K. Attah, I. A. Hinne, A. Abdulai, K. Addo-Osafo, E. L. Appiah, M.-M. Osei, Y. A. Afrane

**Affiliations:** Department of Medical Microbiology, University of Ghana Medical School, College of Health Sciences, University of Ghana, Korle-Bu, Accra, Ghana

**Keywords:** *Plasmodium falciparum*, *Schistosoma mansoni*, *Schistosoma haematobium*, Soil-transmitted helminths, Ghana

## Abstract

**Background:**

Infections of *Plasmodium* species, *Schistosoma* species and soil-transmitted helminths (STH) inflict a significant burden on children mostly in deprived communities in Ghana. Despite the deployment of malaria vector control and the annual Mass Drug Administration by National Control Programmes, these infections still pose major public health concerns in Ghana. Some remote communities which are hard-to-reach are not covered by MDA campaigns which is a major challenge to meeting elimination targets. Adequate data is necessary for formulating policies and strengthening interventions to mitigate transmission. This study assessed the infection burden of *Plasmodium*, *Schistosoma* species and STH infections among school children in communities in Southern and Northern Ghana.

**Method:**

School children living in communities in Southern (Ada Foah, Pediatorkope, Tuanikope) and Northern (Kpalsogu) Ghana were enrolled in a cross-sectional study. A total of 493 (241 males and 252 females) school children aged (2–15 years) were enrolled in the study. Stool samples were collected to screen for *Schistosoma mansoni* and STH infections using the formol-ether concentration technique and urine samples were also collected to screen for *S. haematobium* using the routine urine examination method. *Plasmodium* parasitaemia was determined from thick and thin finger-prick blood samples.

**Results:**

Overall, the prevalence of *P. falciparum*, *S. mansoni*, *S. haematobium*, *Trichuris trichiura* and hookworm infections were 17.2% (95%CI 12.8–19.7), 22.6% (95%CI 25.2–32.7), 1.6% (95%CI 0.89–5.2), 1.2% (95%CI 0.78–4.8) and 1.2% (95%CI 0.78–4.8) respectively. *Plasmodium falciparum* infection was generally widespread in all the study sites with Ada Foah recording the highest prevalence (35.3%) and Kpalsogu recording the lowest (5.8%). *Schistosoma mansoni* was present in only two Southern communities with Tuanikope recording the highest prevalence of 70.3% as against 51.5% recorded in Pediatorkope. A total of 4.5% (95% CI 2.82–10.8) of the children were co-infected with *P. falciparum*, *Schistosoma* species and STHs. This occurred only in the Southern communities; of which combination of *P. falciparum* and *S. mansoni* were predominant (1.4%).

**Conclusion:**

A relatively low burden of parasites co-infection among children only in the Southern communities was detected. However, there were a high prevalence of single infections of *P. falciparum* and *S. mansoni* in those communities. Control measures for the helminths needs to be restarted in the island communities with a high burden of *S. mansoni* infections and that of Plasmodium needs to be scaled up in Ada Foah where *P. falciparum* infections were high.

## Background

Infections of *Plasmodium* species, *Schistosoma* species and soil-transmitted helminths (STHs) are the leading parasitic infections with a considerable disease burden in pre-school and school-going children particularly in Sub-Saharan Africa [1–3]. *Plasmodium* infection and Neglected Tropical Diseases (NTDs) are reported to affect millions of lives in developing countries owing to their great association with poor sanitary conditions, unsafe water sources for domestic activities and low socio-economic status [4, 5]. The distribution of these diseases often overlaps due to various conditions that favour multiple parasitic species survival and transmission [6].

Malaria caused by the *Plasmodium* parasites is endemic and perennial in all parts of Ghana with seasonal variations more pronounced in the northern part of the country. *Plasmodium falciparum* accounts for over 95% of malaria infections of about 4.5 million morbidity [7, 8].

Ghana is among the most schistosomiasis-burdened countries in sub-Saharan Africa with two main human species, *Schistosoma mansoni* and *S. haematobium* causing the disease in various parts of the country where there are freshwater bodies that support the snail vectors [9]. In some communities along the Volta basin in Southern Ghana, prevalence of *S. mansoni* is reported to be as high as 80–90% [10]. A systematic review showing the prevalence, types, geographical and population distribution of soil-transmitted helminths in Ghana over a 10-year period of 2000–2018, identified hookworms, *Ascaris lumbricoides* and *Trichuris trichiura* as the most widespread STHs infections [11]. These infections affect most people living in poor sanitary areas where children, pregnant women and farmers are vulnerable to these infections. The prevalence of STHs in most endemic communities in Ghana has reduced significantly over the years since the scale up of MDA campaigns in some areas [10].

Children co-infected with these parasites develop below optimal and they have increased susceptibility to other infections. They have reduced learning ability which results in poor school achievements [12, 13]. Poly-parasitism acts in multiple ways to adversely affect human health through immune suppression, nutrient malabsorption, anorexia, and haemolysis.

In Ghana, these infections are a major health problem among school children due to their exposure to these parasites in their daily activities which leads to poor cognitive development, retarded growth, general ill health and eventually death [14, 15].

Co-infections of *P. falciparum*, *Schistosoma* species and STHs have been reported from various epidemiological settings in Africa [16]. However, most of these parasitic infections are studied individually thus, data on prevalence, morbidity and mortality associated with multiple parasitic infections in Ghana are limited. More so, some communities in Ghana that are hard-to-reach such as the island communities are not covered by MDA campaigns which is a major challenge to meeting elimination targets.

Knowledge on the extent of parasite co-infections in high-risk groups will serve as a guide in developing sound integrated intervention strategies to reduce the burden of these diseases and co-morbidity. This study was therefore conducted to assess the infection burden of *Plasmodium*, *Schistosoma* species and STH among school children living in communities in Southern and Northern Ghana.

## Materials and methods

### Study communities

**Fig. 1:**
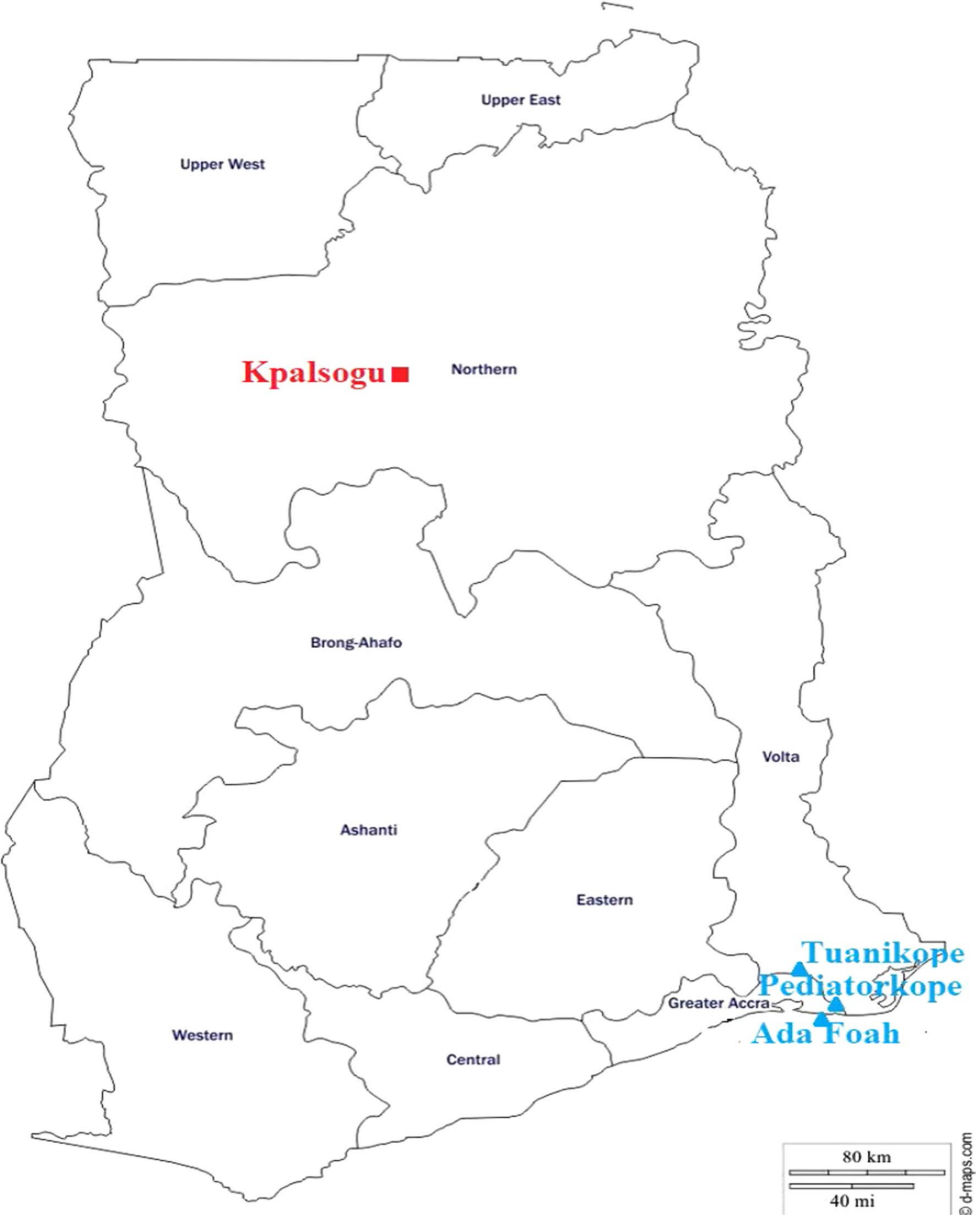
Map of Ghana showing the study sites

The study was carried out in three (3) Southern communities (Tuanikope, Pediatorkope and Ada Foah) all in the Ada East District of the Greater Accra region of Ghana and one (1) Northern community (Kpalsogu) in the Kumbugu District of the Northern region of Ghana (Fig. 1).

Tuanikope (5° 48′ N, 0° 37′ E, 3 m above sea level) and Pediatorkope (5° 49′ N, 0° 37′ E, 3 m) are island communities on the Volta River. The inhabitants are mainly engaged in fishing and rural subsistence farming. Malaria transmission occurs all year round with seasonal peaks reaching high in the rainy season as habitats for vectors increases. These rural communities lack basic social amenities and access to potable water, and thus, depend on river water for domestic chores increasing the risk of helminths infections. These two island communities lie in the coastal savannah ecological zone in Ghana which has a bimodal rainfall pattern. The main rainy season is from April to June and the main dry season is from January to March.

Ada Foah (5° 45′ S, 6° 00′ N, 3 m above sea level) is a coastal community that borders the Volta River to the east. The majority of the inhabitants are engaged mainly in fishing and vegetable farming. Malaria transmission occurs throughout the year as ponds and wells in vegetable farms sustain the breeding of vectors. Intense malaria transmission occurs in the rainy season (March–November). Ada Foah is also found in the coastal savannah zone of Ghana.

Kpalsogu (9° 34′ N, 1° 01′ W, 124 m above sea level) is a farming community in the Kumbungu district in the Northern region of Ghana which lies within the Sahel savannah ecological zone in Ghana with a unimodal rainfall pattern. The rainy season is from May to November and the dry season from December to April. The community has a Dam used for irrigational farming and domestic purposes and also lacks basic amenities which contributes to malaria and helminths transmission.

Each of the four (4) communities has only one basic school which was chosen for the study. The study was carried out between April and May 2019 among 493 school children from four (4) primary schools. Every primary school child who was willing to be part of the study and whose parents provided informed consent was considered for the study.

### Study design and population

A cross-sectional study was undertaken to determine the prevalence and intensity of *Plasmodium* parasites, *S. mansoni*, *S. haematobium* and STHs infections among school children in the study sites. Demographic data including the age and gender of the study participants were recorded.

### Recruitment and sample collection

All primary school-going children aged 3–15 years, in each of the study communities were requested to be part of the study after explaining the purpose of the study to them and their parents/guardians. Those who consented were recruited from each class and signed an assent form with their parents. Each child was screened for infection with Schistosoma (stool and urine), STHs (stool) and Plasmodium (blood).

### Stool collection and parasite determination.

Fresh stool samples in containers labelled with unique identification codes were collected from the school children in May, 2019. The stool samples were taken to the Parasitology Laboratory of the Medical Microbiology Department, University of Ghana Medical School. The formol-ether concentration technique was used to analyze the samples. Briefly, one (1) gram of each stool sample was emulsified in 10 ml of 10% formalin solution in a centrifuge tube using an applicator stick. The suspension was then strained through two layers of gauze into another centrifuge tube. More of the 10% formalin solution was added to the strained suspension to bring the volume to 10 ml. Three (3) millilitres of ether was then added to the suspension, the tube vigorously shaken for 10 s and the sample centrifuged for 3 min at 1000 rpm. The ether-faecal debris and formalin were decanted in a single movement. The sediment was deposited on a clean slide and examined microscopically for schistosome and STH parasite eggs. Infection intensities were classified into light, moderate, and heavy egg counts of 1–99 epg (egg per gram), 100–399 epg and > 400 epg respectively for *S. mansoni*, and 1–999 epg, 1000–9999 epg and > 10,000 epg respectively for STHs [16].

### Urine collection and parasite determination

Each of the study participants was given a labelled 50 ml wide-mouth screw cap container to provide, at least, 15 ml of urine on the day of screening. About 1 ml of 10% formalin was added to each urine sample and transported to the Parasitology Laboratory of the Medical Microbiology Department, University of Ghana Medical School for examination.

Each urine sample was homogenized, and 10 ml transferred into an unused 15 ml centrifuge tube. The samples were centrifuged at × 1000 rpm for 5 min, the supernatant carefully decanted, and the deposits examined for schistosome eggs using the ×10 objectives of the light microscope. The intensity of infection was classified as light when the egg count was less than 50 eggs/10 ml of urine and heavy when egg counts are more than 50 eggs per 10 ml.

### Screening of school children for malaria parasites

Finger prick blood samples were collected after cleaning the finger surface using an alcohol swab. Thick and thin blood smears were prepared with the latter fixed with methanol and stained with 10% Giemsa. Plasmodium parasite densities were estimated from thick smears under the light microscope using the ×100 objective to count the number of parasites per 200 white blood cells (WBC) from the thick smears. The counts of *P. falciparum* were converted to the number of parasites per μl of blood, assuming a standard WBC count of 8000/μl. Thin blood smears were examined for Plasmodium species identification.

### Data analysis

Data were entered into Microsoft Excel 2013 spreadsheets, checked for entry errors, and analyzed using STATA version 15 software (StataCorp. 2017. Stata Statistical Software: Release 15. College Station, TX: StataCorp LLC). The prevalence of single and multiple species of parasites was assessed and classified according to gender and age groups. The Fisher’s exact test was used to assess differences in infection rates of single and double or triple parasitic infections according to sex, age and study community. Logistic regression analysis was applied to investigate whether gender and age were significantly associated with the *S. mansoni*, *P. falciparum* and hookworm infections.

## Results

### Background characteristics

A total of 493 primary school children aged 2–15 years were recruited in this study. Slightly more than half (51.12%) of the participants were females with the majority (47.67%) falling within the 6–10 years age group. The community, age and sex distribution of the study participants are as shown in Table 1.

**Table 1:** Demographic characteristics of study participants

Background characteristics	Frequency (n)	Percentage (%)
*Schools*		
Ada Foah R/C	184	37.32
Tuanikope D/A	64	12.98
Pediatorkope D/A	125	25.35
Kpalsogu Zion	120	24.35
*Sexes*		
Male	241	48.88
Female	252	51.12
*Ages*		
2–5	86	17.44
6–10	236	47.67
11–15	171	34.89

### Prevalence and coinfections pattern of parasites

Overall, 188/493 (38.1%, 95% CI 41.2–47.4) harboured at least one parasitic infection.

Table 2 shows a multivariate analysis of the prevalence pattern of *P. falciparum*, *S. mansoni* and *S. haematobium*. Out of the 493 children screened for malaria parasites, 85 (17.2%, 95% CI 12.8–19.7) were positive for *P. falciparum* (none of the children harboured other *Plasmodium* species). Prevalence was significantly high in children from Ada Foah 65 (35.3%, *p* < 0.001, 95%CI 8.25–14.7) than those from other communities with Kpalsogu recording the least 7 (5.8%).

**Table 2:** A multivariate analysis showing the prevalence pattern of *P. falciparum*, *S. mansoni* and *S. haematobium*

	N	*P. falciparum* infection				*S. mansoni* infection				*S. haematobium* infection			
		n (%)	aOR	95%CI	*p*	n (%)	aOR	95%CI	*p*	n (%)	aOR	95%CI	*p*
Total	493	85(17.2)		12.8–19.7		97(22.6)		25.2–32.7		8(1.6)		0.89–5.21	
*Sites*													
Ada Foah	184	65(35.3)			** < 0.001**	0(0)			** < 0.001**	5(2.7)			0.197
Pediatorkope	125	9(7.2)		8.25–14.7		52(51.5)				3(2.4)			
Tuanikope	64	4(6.3)				45(70.3)				0(0)			
Kpalsogu	120	7(5.8)				0(0)				0(0)			
*Sex*													
Male	241	34(14.1)	1.41	0.86–2.30	0.072	54(23.1)	0.74	0.47–1.18	0.185	4(1.6)	1.28	0.25–6.53	1.000
Female	252	51(20.2)				43(18.2)				4(1.7)			
*Age*													
> 6	86	7(8.1)	1.16	1.07–1.26	**0.004**	11(12.9)	1.10	1.02–1.19	0.095	0(0)	1.39	1.01–1.91	0.504
6–10	236	37(15.7)				48(20.8)				4(1.7)			
11–15	171	41(24.0)				38(24.8)				4(2.3)			

*P* =* p*-value (*p* < 0.05 is statistically significant, p > 0.05 is statistically not significant)

There was a significant difference observed in the prevalence of *P. falciparum* according to age groups (*p* = 0.04, 95% CI 0.07–1.26) with the highest 41(24.0%) recorded in the older age group of 11–15 years followed by age group 6–10 years 37 (15.7%) and lowest in age group 2–5 years 7 (8.1%). *P. falciparum* prevalence showed no significant difference according to gender (*p* = 0.072) although females recorded a higher prevalence of 51 (20.2%).

The overall prevalence of *S. mansoni* in the study was 97 (22.6%, 95% CI 25.2–32.7). *Schistosoma mansoni* was present in only two communities with Tuanikope recording a higher prevalence 45 (70.3%) as compared to Pediatorkope 52 (51.5%). A significant difference in the prevalence of *S. mansoni* was observed between the study communities (p < 0.001, 95% CI 25.2–32.7). Males were more infected 54 (23.1%), than females 43 (18.2%) and a high proportion of infection rate was observed according to age groups. However, no significant difference was observed according to gender and age (Table 2).

The majority of the children infected with *S. mansoni* (89%) had light infections (1–100 epg).

The prevalence of *S. haematobium* in the communities ranged from 0 to 2.7%. Out of the 493 participants, 8 (1.6%, 95% CI 0.89–5.2) were positive for *S. haematobium*. The observed difference in the prevalence between the study communities was not statistically significant. Infection with *S. haematobium* was not gender or age dependent. However, there was an increasing trend with increasing age, with the highest prevalence of 2.3% recorded for the age group 11–15 years (Table 2). The egg count of participants ranged from 2 to 106 eggs/10 ml of urine with only 1 child harbouring a heavy infection.

Hookworm and *T. trichiura* were the only STHs detected albeit with low prevalence (Table 3). The overall hookworm prevalence which was 1.2% (95% CI 0.78–4.8) was recorded only in Pediatorkope and Tuanikope with no significant differences according to the site, gender, and age Prevalence of *T. trichiura* was also 1.2% (95% CI 0.78–4.8) and this was recorded only in Ada Foah (Table 3).

**Table 3:** A multivariate analysis showing the prevalence pattern of Hookworm and *Trichuris trichiura*

	N	Hookworm infection				*Trichuris trichiura* infection			
		n (%)	aOR	95% CI	*p*	n (%)	aOR	95% CI	*p*
Total	493	5(1.2)		0.78–4.8		5(1.2)		0.78–4.8	
*Site*									
Ada Foah	184	0(0)			0.24	5(2.7)			0.09
Pediatorkope	125	3(2.4)				0(0)			
Tuanikope	64	2(3.1)				0(0)			
Kpalsogu	120	0(0)				0(0)			
*Sex*									
Male	241	3(1.2)	0.92	0.13–6.36	0.64	2(0.8)	0.92	0.13–6.36	1.00
Female	252	2(0.7)				3(1.2)			
*Age*									
> 6	86	2(2.3)	0.82	0.59–1.14	0.58	0(0)	0.74	0.67–1.84	0.72
6–10	236	1(0.4)				3(1.3)			
11–15	171	2(1.2)				2(0.85)			

Co-infections in the study participants was low. The prevalence of at least two parasite species in the study participants put together was 22 (4.5%, 95% CI 2.82–10.8) (Table 4). Most of these parasite co-infections predominantly occurred in children from Pediatorkope 59% (13/22), where there was no MDA at time of the sample collection. However, with Kpalsogu, where there was ongoing MDA, no co-infections was recorded. Table 4 shows the prevalence of co-infections, stratified by communities, sex and age among the 493 primary schoolchildren. The co-infections between *S. mansoni* and *P. falciparum* 6 (1.4%) was the most common, followed by co-infections of *S. haematobium* and *P. falciparum* 4 (0.8%). The age group, 6–10 years showed the highest prevalence of co-infection 8 (3.4%) and males also showed a higher prevalence of co-infection.

**Table 4:** Prevalence of co-infections, stratified by communities, sex and age among 493 primary schoolchildren

Variable	N	*P. falciparum* + *S. mansoni*		*P. falciparum* + *S. haematobium*		*P. falciparum* + *T. trichiura*		*S. mansoni* + *S. haematobium*	
		n (%)	*p* (95% CI)	n (%)	*p* (95% CI)	n (%)	*p* (95% CI)	n (%)	*p* (95% CI)
*Site*									
Ada Foah	184	5 (2.7)	0.94 (0.72–1.41)	3 (1.6)	0.54 (1.42–2.34)	2 (1.09)	0.09 (0.56–1.93)	0(0)	0.09 (0.86–2.04)
Pediatorkope	125	0 (0)		1 (0.9)		0 (0)		3 (2.9)	
Tuanikope	64	1(1.6)		0(0)		0 (0)		0(0)	
*Sex*									
Male	241	4(1.9)	0.48 (1.14–2.1)	1(0.4)	0.23 (1.42–2.34)	0(0)	0.09 (0.56–1.93)	3(1.2)	0.08 (1.24–3.36)
Female	252	2 (0.9)		3 (1.2)		2 (0.9)		0(0)	
*Age*									
< 6	86	1(1.6)	0.78 (0.83–3.52)	0(0)	0.16 (0.86–2.81)	0(0)	0.10 (1.12–2.36)	0(0)	0.09 (1.16–2.86)
6–10	236	2 (0.9)		2 (0.9)		2 (0.9)		2 (0.9)	
11–15	171	3 (1.7)		2(0.9)		0(0)		1(0.6)	

The prevalence of co-infections did not differ significantly according to age and gender.

## Discussion

The burden of malaria, schistosomiasis, and STH infections particularly among children is a major public health problem in Ghana where co-infections in a single host do sometimes occur. Even though these infections co-exist, most studies in Ghana focus on individual infections hence data on the co-morbidity of these infections remains limited. Understanding the epidemiology of these infections and their co-infections is an important finding to support the design of integrated disease control strategies. The results from the study demonstrated that *P. falciparum*, *S. mansoni*, *S. haematobium* and STH infections (Hookworm and *T. trichiura*) are prevalent in school children in communities in Southern and Northern Ghana and co-infections of these parasites were detected at a relatively low rate.

It was found in the study that *P. falciparum* infections were present in school children from all study communities and were highest among children in Ada Foah and infection burden lowest in Kpalsogou. Kpalsogou has been under Indoor Residual Spraying (IRS) implementation since 2008 and together with the Long-Lasting Insecticide Nets (LLINs) coverage of > 70% in the area, has contributed to reduction in the burden of malaria in the community. There has been no IRS implementation in Ada Foah and the two island sites of Pediatorkope and Tuanikope. This highlights the gains from the current vector control interventions which needs to be sustained and probably expanded to other endemic areas to further reduce malaria burden. The total prevalence of *P. falciparum* infection in school children in this study was moderate which corroborates with infection rates reported in other studies among school children in Southern Ghana [13]. Logistic regression analysis showed an increased malaria risk for older children which was unexpected as infection burden is expected to decrease with increasing age when children gradually develop some degree of immunity against the Plasmodium parasite due to repeated infections. A similar observation was reported in a study in the Democratic Republic of Congo which found a comparably high *P. falciparum* infection rate among the oldest age group and a high risk of infection with an increase in age [1].

The study also found a high prevalence of *S. mansoni* infection in the Southern communities occurring only in the island sites of Tuanikope and Pediatorkope. This could be attributed to the halt of MDA campaigns in these hard-to-reach island communities for almost a decade (as of September 2019) contributing to high infection rates. Furthermore, these communities lack basic social amenities and unavailability of potable water as participants depend solely on water from the river for domestic purposes thus exposing them to infections. Patterns of *S. mansoni* infection rate showed no significant variation by age and gender since both sexes in these island communities engage in water-related activities which put them at equal risk of infection. The study found a relatively low level of *S. haematobium* infection among participants only in the southern communities. Patterns of *S. haematobium* infection did not differ significantly between communities, gender, and age, owing perhaps to the relatively low levels of infection rate.

The STH infections burden was low in this study with only Hookworm and *T. trichiura* detected at low levels in the southern communities, but none in the northern community due to the sustained roll out of MDA campaigns in this community (Kpalsogu). Previous studies in Ghana reported prevalence of hookworm infection ranges from 3.2 to 10% [17, 18]. The active periodic deworming exercise among school children in some endemic communities in Ghana, targeting intestinal helminths and increased awareness through education, might have been responsible for these low levels of prevalence [13]. The MDA in addition to improved sanitation, access to adequate water supply and proper health care will help significantly, to reduce the burden of STH infection.

The proportion of children with co-infection of parasites in the study was low with children from the island communities (Pediatorkope and Tuanikope) being the most affected. The co-infection of *S. mansoni* and *P. falciparum* was the most common. The co-endemicity of these parasites and their vectors coupled with poor sanitary conditions in these communities facilitates transmission of the parasites and a consequent co-infection of these parasites in a single individual. Co-infections of *P. falciparum* and helminths have clinical implications. Previous studies have clearly documented the relationship between intestinal helminth infections, polyparasitism and cognitive functions, growth, and malnutrition among school children. Children with multiple parasitic infections especially those with heavy infection intensity tend to experience more severe cognitive outcomes and other health problems such as malnutrition than children with only one helminth infection [19, 20]. Studies suggest that co-infection of *P. falciparum* with hookworms or schistosomes tend to exacerbate anaemia and malnutrition morbidities among school children [15, 21]. The total prevalence of *P. falciparum* and *S. haematobium* co-infection occurred at a very low level. This is consistent with findings from a similar study in Southern Ghana which also found a relatively low rate of co-infection of *P. falciparum* and *S. haematobium* [22]. An important limitation of the current study is the use of only microscopy as a diagnostic method which could also have accounted for the relatively low parasite detection rate as it was possible to miss light infections.

## Conclusion

The study found high burden of single infections of *S. mansoni* and *P. falciparum* among primary school children living in Southern communities of Ghana. However, parasites co-infection among the children was relatively low recorded only among children from the Southern communities. We recommend expansion of the vector control strategies IRS and LLINs distribution, scaling up of MDA campaigns and provision of basic social amenities to these Southern communities which are hard- to- reach to enhance reduction of infection burden in Ghana.

## Data Availability

All authors confirm that the data supporting the findings of this study are available within the article. All datasets generated and/or analysed during the current study are available from the corresponding author on request. Data request can be made through the corresponding author’s email.
